# Fucoxanthin Inhibits the NMDA and AMPA Receptors Through Regulating the Calcium Response on Substantia Gelatinosa Neurons of the Trigeminal Subnucleus Caudalis in Juvenile Mice

**DOI:** 10.1155/np/2553040

**Published:** 2025-01-31

**Authors:** Nhung Ha Thuy Le, Seon Ah Park, Yu Mi Kim, Dong Kuk Ahn, Won Jung, Seong Kyu Han

**Affiliations:** ^1^Department of Oral Physiology, School of Dentistry and Institute of Oral Bioscience, Jeonbuk National University, Jeonju, Republic of Korea; ^2^Faculty of Odonto-Stomatology, Hue University of Medicine and Pharmacy, Hue University, Hue, Vietnam; ^3^Department of Oral Physiology, School of Dentistry, Kyungpook National University, Daegu, Republic of Korea; ^4^Department of Oral Medicine, School of Dentistry and Institute of Oral Bioscience, Jeonbuk National University; Research Institute of Clinical Medicine of Jeonbuk National University—Biomedical Research Institute of Jeonbuk National University Hospital, Jeonju 54896, Republic of Korea

**Keywords:** AMPA receptors, calcium, fucoxanthin, glutamate excitotoxicity, NMDA receptors, patch-clamp, substantia gelatinosa neuron

## Abstract

Glutamate excitotoxicity is considered as the etiology of stroke and neurodegenerative diseases, namely, Parkinson's disease (PD), Alzheimer's disease (AD), and others. Meanwhile, substantia gelatinosa (SG) neurons of the trigeminal subnucleus caudalis (Vc), a pivotal site in regulating orofacial nociceptive transmission via Aδ and C primary afferent fibers, majorly utilize glutamate as the principal excitatory neurotransmitter. Fucoxanthin (FCX), a carotenoid pigment extracted from brown seaweed, possesses various pharmaceutical properties including neuroprotective effect in multiple neuronal populations. To date, the direct activity of FCX on the SG of the Vc has not been extensively clarified. Consequently, we investigated the effect of FCX on excitatory signaling mediated by ionotropic glutamate receptors (iGluRs), using the patch-clamp technique recorded from SG neurons of the Vc. Here, FCX directly acted on glutamate receptors independent of voltage-gated sodium channel and γ-aminobutyric acid (GABA)_A_/glycine receptors in the voltage-clamp mode. Specifically, the *N*-methyl-D-aspartic acid (NMDA)- and α-amino-3-hydroxy-5-methyl-4-isoxazolepropionic acid (AMPA)-induced responses but not the kainic acid receptor (KAR)-mediated response were suppressed by FCX in standard extracellular solution. Additionally, the inhibitory effect of FCX on NMDA currents was repeatable and concentration-dependent. The FCX blockade of NMDA-mediated excitotoxicity was associated with the modulation of Ca^2+^ response without affecting Na^+^ ions. The Ca^2+^-dependent fluorescence intensity of brain slice was reduced in the presence of FCX. Notably, FCX significantly attenuated the spontaneous firing activity of SG neurons. Altogether, these results reveal that FCX may protect SG neurons against glutamate excitotoxicity via primarily regulating Ca^2+^ response, thereby inhibiting the excitatory signaling induced by NMDA and AMPA receptors (AMPARs).

## 1. Introduction

It is firmly perceived that peripheral nociceptive signals from cranial orofacial region are transmitted centrally via Aδ and C nerve fibers and processed primarily by neurons in the spinal trigeminal subnucleus caudalis (Vc) nucleus of the brain stem [1]. Within this modulatory system, lamina II (substantia gelatinosa [SG]) neurons are known as the major second-order sensory neurons and project their axon terminals to the SG and surrounding laminae [2]. As a result, alterations in SG neuronal excitability through the primary afferent and local interneurons play a pivotal role in the central sensitization of pathological pain [3].

Typically, the majority of synapses originated from SG neurons use glutamate as the principal excitatory neurotransmitter [4]. It has been observed that excessive release of extracellular glutamate might trigger abnormal excitatory neurotransmission followed by neuronal damage or death [5]. Hence, the glutamate-induced excitotoxicity has been hypothesized as the etiology of neurological impairments, including Alzheimer's disease (AD), Parkinson's disease (PD), amyotrophic lateral sclerosis, and cerebral ischemia [6, 7]. Besides, the overstimulation of ligand-gated ionotropic glutamate receptors (iGluRs) leads to an aberrant increase in Ca^2+^ influx, reported in several neuronal populations [8]. The Ca^2+^ dyshomeostasis may initiate various downstream processes, including generation of reactive oxygen species, impairment of endoplasmic reticulum (ER) function, and initiation of protease (calpain) and nitric oxide synthase, thereby inducing apoptotic signaling [9]. Interestingly, due to high Ca^2+^ permeability, neuroprotective therapies have initially focused on blockade of Ca^2+^ entry via *N*-methyl-D-aspartic acid receptors (NMDARs). Therefore, it was subsequently proven that several NMDAR antagonists, such as memantine, selfotel, and others, might protect neurons from glutamate-induced excitotoxicity, offsetting their failure to show efficacy in high-profile clinical trials [10].

In recent years, marine bioactive compounds, such as carotenoids, are considered as novel medicinal alternatives for human health [11]. Fucoxanthin (FCX) (C_42_H_58_O_6_), a xanthophyll-type carotenoid, is widely distributed in edible brown seaweed, including *Undaria*, *Sargassum*, *Laminaria*, *Eisenia*, or Hijikia [12]. It is noteworthy that FCX has become a promising pharmaceutical constituent without significant toxicity effects on human metabolite system [12]. Therefore, recent evidences have suggested that FCX exhibits enormous physiological and beneficial biological properties, including anticancer, antiobesity, and antihypertension [13, 14]. Based on the second most effective antioxidant activity, this carotenoid has also been a therapeutic agent for oxidative stress-related diseases in various in vitro models, such as amyloid-β42-treated microglia cells, ferric nitrilotriacetate-treated hepatic cells, and UV-treated fibroblast cells [15]. Additionally, FCX has been reported to be an anti-inflammatory, hepatoprotective, cardioprotective, cerebrovascular, and neuroprotective factor [16, 17]. Hence, FCX exerts anti-AD effect by suppressing acetyl/butyrylcholinesterase, monoamine oxidase, and Aβ levels [12, 18] as well as anti-PD effect by attenuating α-synuclein expression, oxidative stress, and gliosis–synuclein expression [19]. Such activities are consistent with the ability of FCX to readily penetrate the blood–brain barrier to treat neurological dysfunction [20]. Notably, this carotenoid produced a protective effect from glutamate excitotoxicity, which was manifested by the activation of parkin-mediated mitophagy on retinal ganglion cells [21]. Likewise, the neuroprotective role of astaxanthin, another natural xanthophyll similar to FCX, is involved in the inhibition of intracellular Ca^2+^ rise induced by glutamate and the suppression of NMDAR mRNA expression [22–24]. Consequently, it is of interest to evaluate whether FCX could protect SG neurons of the Vc from glutamate toxicity.

FCX has exhibited neuroprotective effects in vitro and has been successfully applied in in vivo animal models. The oral intake of FCX could result in the effective concentration of fucoxanthinol, a main metabolite of FCX, in the brain, and prevent neuronal injury in the model of traumatic brain injury [25]. Furthermore, FCX has shown to promote neurite outgrowth activity (15.7%–31%) at low concentrations (0.01–2 µM), suggesting that this marine carotenoid may a powerful neurotrophic molecule [26]. Due to the involvement of multiple pathogeneses in neurodegenerative disorders, including amyloid protein aggregation, oxidative stress, neuroinflammation, neuronal loss, and neurotransmission, FCX with long-term treatment (normally daily treatment for several days) is a one-compound multiple-target pharmaceutical and nutraceutical candidate to manage neurological dysfunctions [20]. However, the concentration of FCX relevant in in vivo neuroprotection is relatively high, and its low bioavailability has restricted the clinical application of this pigment in the treatment of neurodegenerative diseases. Although several strategies have been offered to improve preventive benefits for neurological conditions, such as combining FCX with dietary oil or milk, or loading it onto nanoparticles, there was only one clinical study about the protective activity of FCX on obesity reported in the last 5 years [27]. This suggests that further studies should develop brain-targeted oral delivery systems to enhance the translational research into human subjects. In this regard, FCX-loaded nanoparticles with casein and FCX-casein coated with chitosan were synthesized and increased the utilization of FCX in the food industry [28]. Moreover, poly lactic-co-glycolic acid-block-polyethylene glycol-loaded FCX (PLGA-PEG-Fuc) nanoparticles with diameter of ~200 nm and negative charge were developed and found to facilitate the bioavailability and efficacy of FCX in treating AD when injected intravenously [29]. Since the bioavailability of fucoxanthiol is around 90 times higher than FCX, it could produce the active metabolites of FCX as antineurodegenerative disease drugs or to directly use FCX as a natural prodrug of the same active metabolites. According to the NIH ClinicalTrials.gov database, four human trials of FCX are presently enrolling for the future [20]. Further evaluation in human clinical tests may make a crucial progress in developing FCX or its bioactive metabolites as functional food ingredients or pharmaceutical treatments for neurodegenerative diseases.

Hence, the present study focused on the beneficial effects of FCX on SG neurons of the Vc and explored its underlying mechanisms observed in glutamate neurotoxicity. To investigate the hypothesis about the neuroprotective effect of this xanthophyll on excitotoxicity, we examined the FCX action on the iGluR-mediated excitatory responses of SG neurons in juvenile mice by patch-clamp electrophysiology. We also suggest that further studies involving are essential to assess its possible application in the management of neurological disorders.

## 2. Methods and Materials

### 2.1. Animals

All animal care conditions and experimental procedures involving the use of animals were approved by the Institutional Animal Care and Use Committee of Jeonbuk National University (JBNU 2022-034). Electrophysiological experiments were performed using brain slices prepared from immature ICR mice of either sex (postnatal day 7–20) housed under 12 h light–12 h dark cycle (lights on at 07:00 a.m.) with ad libitum access to food and water.

### 2.2. Brain Slice Preparation

Coronal brain slices were prepared using the same procedure as described in a previous study [30, 31]. In brief, mice were decapitated between 10:00 and 12:00. UTC+09:00 (Universal Time Coordinated). Their brains were swiftly removed and placed in ice-cooled (2–4°C) standard artificial cerebrospinal fluid (ACSF) containing 126 mM NaCl, 2.5 mM KCl, 2.4 mM CaCl_2_, 1.2 mM MgCl_2_, 11 mM D-glucose, 1.4 mM NaH_2_PO_4_, and 25 mM NaHCO_3_ (pH value of 7.3–7.4 was maintained when bubbled with 95% O_2_ and 5% CO_2_). After removing the cerebral hemispheres and cerebellum, the brainstem containing the Vc region was mounted on the chilled stage and prepared into 190–220-μm coronal slices by a microslicer (VT1200S; Leica biosystem, Wetzlar, Germany). These slices were used for recording after a recovery period of 1 h in oxygenated ACSF at room temperature.

### 2.3. Electrophysiology

Each brain slice was relocated to a recording chamber mounted on the stage of an upright microscope (BX51W1; Olympus, Tokyo, Japan). They were fixed with an anchor and continuously superfused at 3–4 mL/min with oxygenated ACSF. The image of the coronal slice was displayed on a video monitor. SG neurons of the Vc were identified at ×10 and ×40 objective magnifications and patched under infrared–differential interference contrast optics. The SG (lamina II) of the Vc was clearly detected as a translucent band along the lateral edge of the coronal slice and just medial to the spinal trigeminal tract.

All electrical measurements were recorded under the conventional voltage-clamp technique (−60 mV) with a high Cl^–^ pipette solution. Patch pipettes with open-tip resistances of 4–6 MΩ were constructed from thin-walled borosilicate capillary glass (PG 52151-4; WPI, Sarasota, FL, USA) on a Flaming/Brown puller (P-97; Sutter Instruments Co., Novato, CA, USA). Patch electrodes were filled with an intracellular solution composed of 140 mM KCl, 1 mM CaCl_2_, 1 mM MgCl_2_, 10 mM 4-(2-hydroxyethyl)piperazine-1-ethanesulfonic acid (HEPES), 4 mM Mg-ATP, and 10 mM ethylene glycol-bis(2-aminoethyl ether)-N,N,N′,N′-tetraacetic acid (EGTA) (adjusted to pH 7.3 with KOH). After gaining a giga-ohm seal, the cell membrane patch was ruptured under a negative pressure by a short suction pulse for the whole cell mode. To record spontaneous neuronal firings, neurons were patched in cell-attached voltage-clamp mode at a holding potential of 0 mV. Signals were sequentially amplified with an Axopatch 200B amplifier (Molecular Devices, San Jose, CA, USA), filtered at 1 kHz, and digitized at 2 kHz. The membrane potential changes were sampled with an Axon Digidata 1550B interface (Molecular Devices, San Jose, CA, USA) connected to a desktop PC. The acquisition and subsequent analysis of the data were performed with Clampex 10.6 software (Molecular Devices, CA, USA), and traces were plotted using Origin 8 software (OriginLab Corp, Northampton, MA, USA). Experiments were performed at room temperature.

To examine the effect of FCX on the firing activities of SG neurons, brain slices were superfused with 60 μM FCX for 3 min. Excitatory neurotransmitter-mediated responses were elicited by the bath application of glutamate (100 μM) and iGluR agonists, including NMDA (30 μM), α-amino-3-hydroxy-5-methyl-4-isoxazolepropionic acid (AMPA; 3 μM), and kainic acid (KA; 10 μM) for 2 min at a perfusion rate of 3–4 mL/min. Standard ACSF solution was applied to record AMPA- and KA-induced current. However, glycine (1 μM) was continuously coapplied in standard ACSF to record NMDA-induced current, as glycine-enhanced NMDA action is associated with a decrease in NMDAR desensitization. To test the postsynaptic effect of FCX, tetrodotoxin (TTX) (a voltage-gated sodium channel blocker; 0.5 μM), picrotoxin (Picro) (a noncompetitive γ-aminobutyric acid (GABA_A_) receptor antagonist; 50 μM), and strychnine (Stry) (a glycine receptor antagonist; 2 μM) were added to the ACSF solution during that experiment to block voltage-gated sodium and chloride channels [32]. Some recordings were conducted in Ca^2+^-free external solution which comprised 126 mM NaCl, 2.5 mM KCl, 1.2 mM MgCl_2_, 1.4 mM NaH_2_PO_4_, 11 mM D-glucose, 25 mM NaHCO_3_, and 2 mM EGTA. Similarly, Na^+^-free ACSF was prepared by replacing equimolar NaCl with a choline chloride to decrease passive Na^+^ influx [33].

### 2.4. Fluorescence Ca^2+^ Imaging

The Fluo-3 AM staining assay was used to image the Ca^2+^ ion uptake of neurons. Stock solutions (1 mM) of Fluo-3 AM (F1241, Thermo Fisher, MA, USA) were prepared by dissolving in dimethyl sulfoxide (DMSO, Sigma-Aldrich). Brain slices were incubated in oxygenated ACSF including 1 μM Fluo-3 AM for 40 min in the dark. All preparation and loading steps were performed at room temperature in foil-wrapped tubes to protect dye fluorescence. Afterward, stained slices were rinsed three times with ACSF, and the incubation was continued for an additional 30 min before imaging. Fluorescent signals were captured at 1-min intervals for 5 min in the absence and presence of FCX (60 μM) using a fluorescence microscope (EVOS M5000, Thermo Fisher, MA, USA). To visualize the variation in the spontaneous activity of Ca^2+^ signals in the application of FCX, the laminae I–II regions over the field of view were selected, and the mean pixel intensity (MPI) at each frame was measured using the EVOS M5000 software (EVOS M5000, Thermo Fisher, MA, USA). The data were converted to a relative scale (*∆F*/*F*_0_, *∆F* = *F* − *F*_0_), where *F* was the mean fluorescence of the laminae I–II region at each frame, and *F*_0_ was the mean baseline fluorescence.

### 2.5. Materials

TTX and AMPA were purchased from Tocris Bioscience (Avonmouth, Bristol, UK). Fluo-3 AM was bought from Thermo Fisher (F1241, Thermo Fisher, MA, USA). The remaining chemicals, such as Picro, Stry hydrochloride, glycine, glutamate, NMDA, AMPA, KA, and chemical ingredients of ACSF were obtained from Sigma-Aldrich (St. Louis, MO, USA), apart from FCX (≥97% HPLC) from MedChemExpress (Princeton, New Jersey, USA). Stock solution of FCX was dissolved in DMSO, aliquoted, and always stored at −20°C in dark. For each experiment, a fresh aliquot of FCX was thawed and diluted (usually 500-fold) in ACSF to the indicated working concentrations. The stock concentration of FCX was 30 mM. The concentration of DMSO in the final solution was less than 0.33%, and this does not affect membrane potential of SG neurons.

### 2.6. Data Analysis

The Mini Analysis software (ver. 6.0.7; Synaptosoft Inc., Decatur, GA, USA) was used to compare the changes in the frequency of spontaneous firings following drug application. An equivalent time course (2 min) was set to compare firing frequencies of the control, FCX application, and washout. The threshold for analysis of action currents was set at twice the root mean square of background noise, and each automatically recorded event was visually verified to be free of artifacts. The relative percentage was determined by the ratio of target response to its control response and multiplying by 100. Analysis of Ca^2+^ signals was completed using the EVOS M5000 software (EVOS M5000, Thermo Fisher, MA, USA).

### 2.7. Statistics

Results were expressed as mean ± standard error of the mean (SEM). The difference between two and more than two groups was evaluated by one-way analysis of variance (ANOVA) with a post hoc Scheffe test. A *p* value of <0.05 was considered as statistical significance. Statistical analyses were performed using Origin 8 software (OriginLab Corporation, Northampton, MA, USA). For electrophysiological data, the number of samples *n* refers to the number of neurons recorded; *N* refers to the number of animals recorded.

## 3. Results

To investigate the effect of FCX on SG neurons, cell-attached and whole-cell voltage-clamp recordings were conducted from 78 SG neurons of the Vc in juvenile mice. There was no remarkable change in the holding current to maintain neurons at −60 mV in the presence of FCX.

### 3.1. Effect of FCX on Spontaneous Firing Activities of SG Neurons

**Figure 1:**
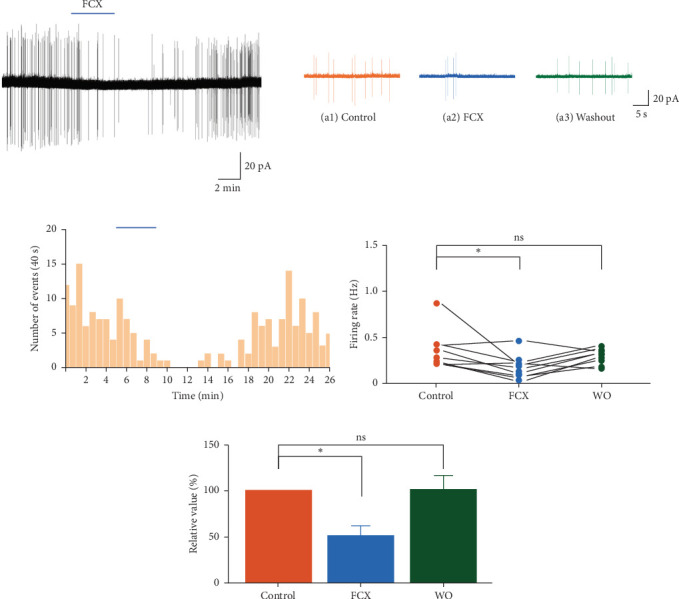
Fucoxanthin (FCX) suppressed the spontaneous firing activities of substantia gelatinosa (SG) neurons. (a) A representative trace of action currents recorded from a SG neuron in the application of FCX (60 μM) in the cell-attached mode. (a_1_−a_3_) Sections of the current trace in (a) show spontaneous firings before, during, and after perfusion of FCX for 30 s, respectively. (b) A spike frequency histogram (bin size 40 s) of the current trace in (a). (c) A line series plot showing firing rate in the presence and washout of FCX. (d) A bar graph showing the firing rate percentages in the presence and washout of FCX. Data are expressed as mean ± standard error of the mean (SEM); *n* = 9; *⁣*^*∗*^*p* < 0.05; ns implicates not significant; one-way analysis of variance (ANOVA).

To assess the impact of FCX on the spontaneous firing activities of SG neurons, recordings were conducted in cell-attached voltage-clamp mode. Perfusion of FCX (60 μM) for 3 min significantly inhibited the frequency of spontaneous neuronal firings (control: 0.36 ± 0.07 Hz; FCX: 0.18 ± 0.05 Hz; washout: 0.32 ± 0.03 Hz; *n* = 9, *N* = 9) (Figure 1a). The time frequency histogram in Figure 1b illustrates a suppression in the frequency of firing activities following FCX application. Consequently, the firing rate of SG neurons was markedly attenuated to 51.6% ± 11.2% in the presence of FCX (*n* = 9, *p* < 0.05, one-way ANOVA) (Figure 1c,d). This finding thus reflects the depressant effect of FCX on the neuronal excitability of SG neurons in the Vc region.

### 3.2. Inhibitory Effect of FCX on Glutamate-Mediated Activation in SG Neurons

**Figure 2:**
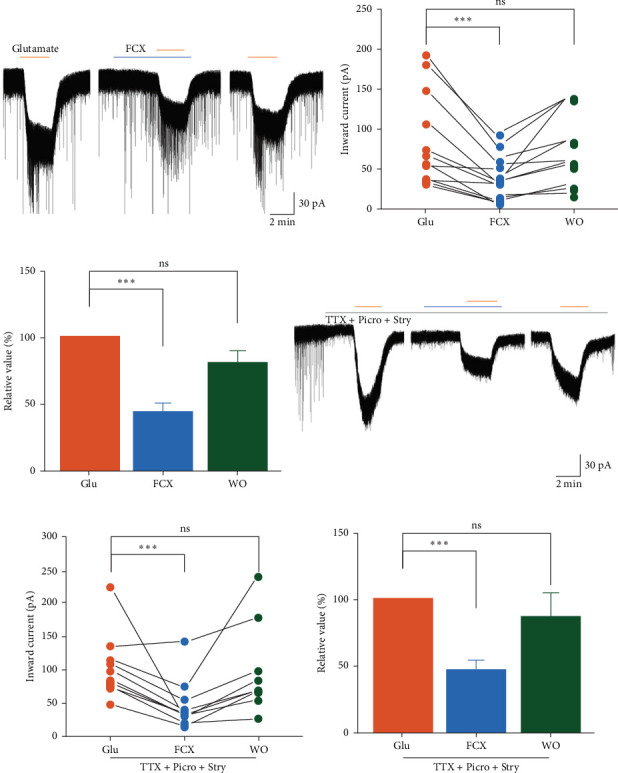
Effects of fucoxanthin (FCX) on glutamate-mediated responses in substantia gelatinosa (SG) neurons. (a, d) Representative traces showing inhibition of glutamate-mediated responses by FCX (60 μM) in the presence of glutamate alone (100 μM) and in the coapplication of tetrodotoxin (TTX, 0.5 μM), picrotoxin (Picro, 50 μM), and strychnine (Stry, 2 μM), respectively. (b, e) Line series plots showing inward currents induced by glutamate, TTX, Picro, and Stry in the absence and presence of FCX. (c, f) Bar graphs showing the mean relative percentage of inward currents induced by glutamate, TTX, Picro, and Stry in the absence and presence of FCX. Data are expressed as mean ± standard error of the mean (SEM); *n* = 12 and 10 in glutamate group and glutamate coapplied with antagonists, respectively; *⁣*^*∗∗∗*^*p* < 0.001; ns implicates not significant; one-way analysis of variance (ANOVA).

Given that glutamate is a ubiquitous excitatory neurotransmitter in the central nervous system (CNS) functioning via ligand-gated ion channels, we checked the modulatory effect of FCX on potential responses induced by glutamate and its subtype agonists. Under the whole-cell voltage-clamp recording, exogenous application of glutamate (100 µM, 2 min) elicited inward currents with a mean amplitude of −85.3 ± 16.7 pA (*n* = 12) (Figure 2a). However, pretreatment of FCX (60 µM) for 3 min significantly reduced the glutamate-mediated responses (FCX: −37.3 ± 7.89 pA, *n* = 12; *N* = 12) (Figure 2b). The mean relative percentage of inward currents induced by glutamate in the presence of FCX was 44.5% ± 6.58% compared to glutamate alone (*n* = 12, *p* < 0.001, one-way ANOVA) (Figure 2c). These effects of FCX were partially reversible, and the amplitude of currents recovered to ~80% of the control value after a 5-min washout.

In the next steps of experiment, the effect of FCX on glutamate-induced response was recorded in the presence of TTX (0.5 μM), Picro (50 μM), and Stry (2 μM) to assess whether FCX directly interacts with iGluRs of SG neurons or involves action potential-modulated mechanism. Under this condition, FCX (60 µM) continued to attenuate the glutamate-mediated response (glutamate: −104 ± 15.6 pA; FCX: −48.6 ± 11.8 pA, *n* = 10; *N* = 10) (Figure 2d,e). Specifically, the mean relative percentage of inward currents mediated by glutamate in the presence of FCX and blockers was 47.1% ± 7.8% of the glutamate alone (*n* = 10, *p* < 0.05, one-way ANOVA) (Figure 2f). This observation suggests the direct effect of FCX on the iGluRs, independent of action potential and GABA/glycine interventions.

### 3.3. Effects of FCX on the Glutamate Receptor Agonist-Mediated Responses in SG Neurons

To characterize the inhibitory effect of FCX on iGluRs of SG neurons, we subsequently measured the activity of their specific agonists, such as AMPA, NMDA, and KA in the presence of FCX. In the absence of FCX, these agonists were applied for 2 min following a 5-min baseline recording. In the presence of FCX, after FCX was perfused within 3 min, these agonists were added into ACSF without removing FCX.

**Figure 3:**
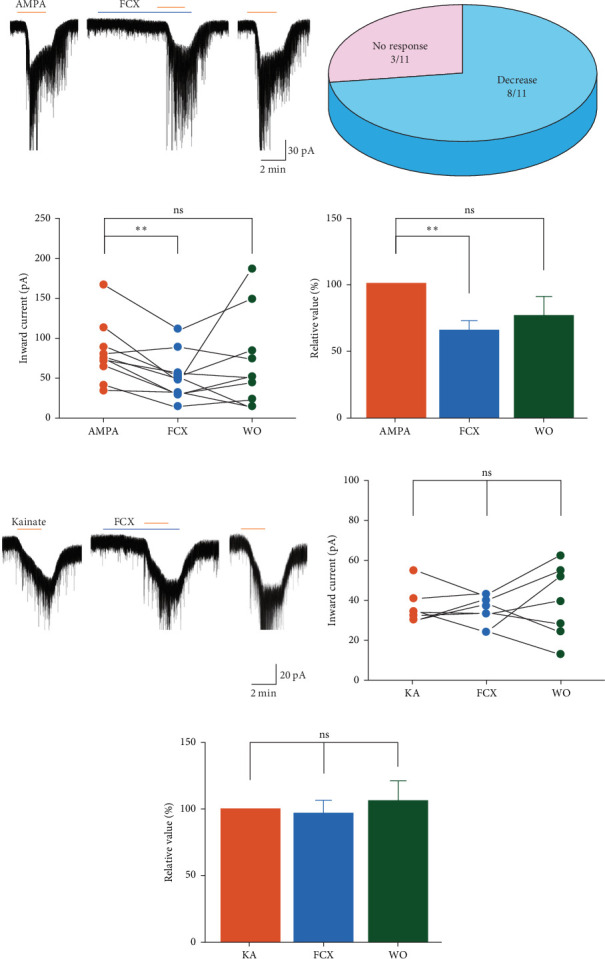
Effects of fucoxanthin (FCX) on α-amino-3-hydroxy-5-methyl-4-isoxazolepropionic acid (AMPA)- and kainic acid (KA)-mediated responses. (a, e) Representative traces showing inward currents induced by AMPA (3 μM) and KA (10 μM) in the absence and presence of FCX (60 μM). (b) A pie chart showing response rate on AMPA-induced inward current by FCX. (c, f) Line series plots showing inward currents induced by AMPA and KA in the presence of FCX and after washout of FCX compared to AMPA and KA alone. (d, g) Bar graphs showing the mean relative percentage of inward currents mediated by AMPA and KA in the presence of FCX compared to AMPA and KA alone. Data are expressed as mean ± standard error of the mean (SEM); *n* = 11 and 7 in AMPA and KA groups, respectively; *⁣*^*∗∗*^*p* < 0.01; ns, not significant; one-way analysis of variance (ANOVA).

In contrast to NMDARs, the activation of AMPA receptors (AMPARs) and KA receptors (KARs) is neither affected by extracellular Mg^2+^ nor dependent on costimulation of the receptor with coagonists (the amino acid glycine or serine) to promote channel opening. Thus, we predicted that FCX had no significant influence on the actions of AMPAR and KAR. To clarify such hypothesis, we applied AMPA and KA in the absence and presence of FCX. Here, in all tested neurons, the bath application of AMPA (3 µM) showed substantial inward currents with a mean amplitude of −75.6 ± 11.5 pA (*n* = 11). Surprisingly, suppression of AMPA-mediated responses by FCX (60 µM) was displayed in 8/11 (72.7%) neurons, as shown in Figure 3a (AMPA: −85.6 ± 13.4 pA; FCX: −48.0 ± 10.2 pA; washout: −77.6 ± 24.6 pA; *n* = 8, *p* < 0.05, one-way ANOVA). On the contrary, the remaining three neurons were recorded unaffected in the presence of FCX (Figure 3b). Collectively, the mean relative percentage of AMPA-induced inward current was reduced to 65.8% ± 7.49% in the presence of FCX (*n* = 11, *N* = 10; *p* < 0.05, one-way ANOVA) (Figure 3c,d).

On the other hands, 3-min exposure of cells with FCX has no remarkable influence on potential responses induced by KA (10 µM) (KA: −42.3 ± 3.33 pA; FCX: −39.4 ± 2.76 pA; *n* = 7; *N* = 7) (Figure 3e,f). Hence, the mean relative percentage of KA-evoked inward current in the presence of FCX was 96.9% ± 10.1% of the control value (*n* = 7, *p* > 0.05, one-way ANOVA) (Figure 3g).

**Figure 4:**
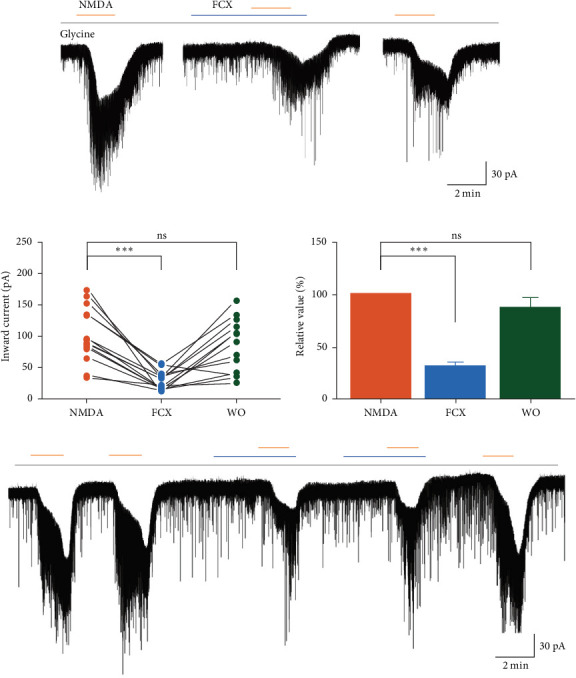
Effects of fucoxanthin (FCX) on *N*-methyl-D-aspartic acid (NMDA)-mediated responses. (a) A representative trace showing inward currents induced by NMDA (30 μM) in the absence and presence of FCX (60 μM). (b) A line series plot showing inward currents induced by NMDA in the presence of FCX and after washout of FCX compared to NMDA alone. (c) A bar graph showing the mean relative percentage of inward currents mediated by NMDA in the presence of FCX and after washout of FCX compared to NMDA alone. (d) The inhibitory effect of FCX on NMDA-mediated response is repeatable. Data are expressed as mean ± standard error of the mean (SEM); *n* = 14; *⁣*^*∗∗∗*^*p* < 0.001; ns, not significant; one-way analysis of variance (ANOVA).

Figure 4a illustrates a clear inward current evoked by NMDA exposure (30 µM), reflecting the activation of NMDARs in the presence of extracellular Ca^2+^. Furthermore, the administration of FCX (60 µM) rapidly and reversibly abolished the NMDA-induced action in SG neurons (NMDA: −102 ± 11.7 pA; FCX: − 29.5 ± 3.84 pA, *n* = 14; *N* = 12) (Figure 4b). Hence, the mean relative percentage of NMDA current in the presence of FCX and FCX washout compared to NMDA alone was 32.1% ± 3.74% and 87.6% ± 9.68%, respectively (*n* = 14, *p* < 0.001, one-way ANOVA) (Figure 4c). Such suppressive effect of FCX on NMDA-induced currents was repeatable, as shown in Figure 4d. In addition, there was no significant difference in the suppression of NMDA currents by FCX between males (29.0% ± 5.69%, *n* = 6) and females (34.4% ± 5.08%, *n* = 8) compared to NMDA alone. All data were collectively analyzed and altogether revealed the inhibitory effect of FCX on NMDAR-mediated responses on SG neurons of the Vc.

**Figure 5:**
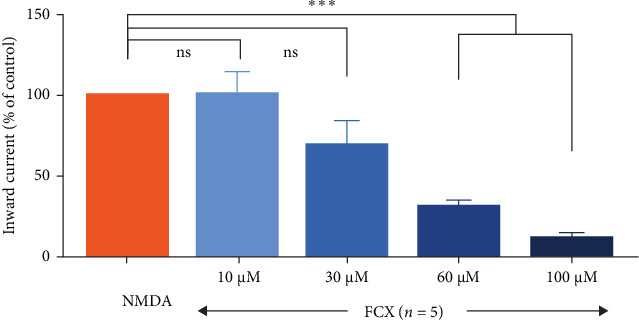
Dose-dependent inhibition of fucoxanthin (FCX) on *N*-methyl-D-aspartic acid (NMDA)-mediated responses. Histogram showing the mean relative percentages of inward currents induced by NMDA (30 μM) in the absence and presence of various concentrations (10, 30, 60, and 100 μM) of FCX. Data are expressed as mean ± standard error of the mean (SEM); *n* = 5 for each concentration except for 60 μM FCX; *n* = 14; *⁣*^*∗∗∗*^*p* < 0.001; ns, not significant; one-way analysis of variance (ANOVA) post hoc Scheffe test.

Next, the activity of FCX on the NMDAR-mediated response was concentration-dependent since bath applications of 10, 30, 60, and 100 μM concentrations of FCX induced a dose-dependent inhibitory effect of NMDA on SG neurons. The mean relative percentages of inward currents by NMDA in the presence of 10, 30, 60, and 100 μM FCX compared to those of NMDA alone were 101% ± 12.8%, 70.5% ± 14.0%, 32.1% ± 3.74%, and 12.7% ± 3.14%, respectively (*n* = 5; *N* = 5) (*p* < 0.001, one-way ANOVA) (Figure 5).

### 3.4. Effects of FCX on the Ca^2+^ Influx Evoked by NMDA Receptors

**Figure 6:**
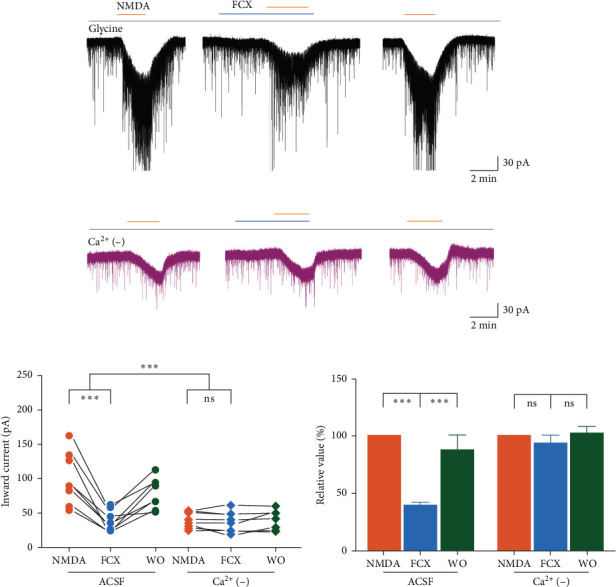
Effects of fucoxanthin (FCX) on *N*-methyl-D-aspartic acid (NMDA)-mediated responses in the absence of extracellular Ca^2+^ in substantia gelatinosa (SG) neurons. (a, b) Representative traces showing inhibition of NMDA-mediated responses by FCX (60 μM) in the presence and absence of extracellular Ca^2+^, respectively. (c) A line series plot showing NMDA-induced inward currents suppressed by FCX in Ca^2+^ containing standard solution compared to extracellular Ca^2+^ lacking solution. (d) A bar graph showing the mean relative percentage of NMDA-induced inward current suppressed by FCX in Ca^2+^ containing standard solution compared to extracellular Ca^2+^ lacking solution. Data are expressed as mean ± standard error of the mean (SEM); *n* = 8; *⁣*^*∗∗∗*^*p* < 0.001; ns, not significant; one-way analysis of variance (ANOVA).

We next investigated the mechanism underlying the modulatory effect of FCX on NMDAR-mediated response on SG neurons. Previous research has indicated that the primary rapid influx of extracellular Ca^2+^ and the delayed Ca^2+^ deregulation are the main causes of glutamate excitotoxicity in some neuronal cultures [34, 35]. Furthermore, provided about the relationship between the biological property of marine products extracted from brown algae (FCX, astaxanthin, fucoidan…) and glutamate-induced Ca^2+^ responses [22, 36], we evaluated the effect of FCX on NMDA-mediated response in the absence of extracellular Ca^2+^. In general, in Ca^2+^-free solution, NMDA superfusion produced a lesser mean amplitude of inward current than that observed in standard ACSF (40.7% ± 3.6% of the control; *n* = 8, *p* < 0.05, paired *t*-test) (Figure 6a,b), suggesting that the bulk of Ca^2+^ influx was primarily responsible for the activation of NMDARs on SG neurons. Notably, FCX failed to block the NMDA-evoked inward currents in the absence of extracellular Ca^2+^ (NMDA: −37.9 ± 4.07 pA; FCX: −36.1 ± 5.24 pA; washout: −39.1 ± 4.97 pA; *n* = 8, *N* = 6) (Figure 6c). The mean relative percentage of inward currents induced by NMDA in the presence of FCX compared to that of NMDA alone was 92.8% ± 5.51% (*n* = 8, *p* > 0.05, one-way ANOVA) (Figure 6d), indicating that the inhibitory effect of FCX on NMDARs was sensitive to Ca^2+^ transients from the external space into the cell.

**Figure 7:**
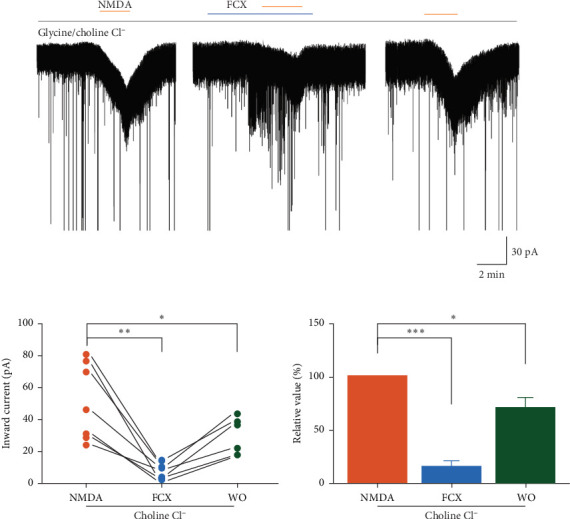
Effects of fucoxanthin (FCX) on *N*-methyl-D-aspartic acid (NMDA)-mediated responses in choline chloride containing solution in substantia gelatinosa (SG) neurons. (a) A representative trace showing inhibition of NMDA-mediated responses by FCX (60 μM) in the presence of extracellular choline chloride. (b) A line series plot showing NMDA-induced inward current in the absence and presence of FCX in choline chloride containing standard solution. (c) A bar graph showing the mean relative percentage of NMDA-induced inward current in the absence and presence of FCX in choline chloride containing standard solution. Data are expressed as mean ± standard error of the mean (SEM); *n* = 7; *⁣*^*∗*^*p* < 0.05, *⁣*^*∗∗*^*p* < 0.01, *⁣*^*∗∗∗*^*p* < 0.001; one-way analysis of variance (ANOVA).

We further probe the efficacy of FCX inhibition of NMDARs in the Na^+^-free ACSF solution to rule out the involvement of Na^+^ influx in FCX activity. Results revealed that when NaCl was equimolarly replaced by choline chloride in the extracellular medium, NMDA perfusion still induced inward currents with a mean amplitude of −51.2 ± 9.14 pA (Figure 7a). Interestingly, the FCX-induced suppression of NMDARs was maintained in the Na^+^-free extracellular solution (FCX: −8.03 ± 1.99 pA; washout: −34.6 ± 5.27 pA; *n* = 7; *N* = 5) (Figure 7b). Under this condition, the mean relative percentage of inward currents evoked by NMDA in the presence of FCX was 16.7% ± 4.18% of the control value (*n* = 7, *p* < 0.001, one-way ANOVA) (Figure 7c). These findings suggest that FCX selectively blocks the Ca^2+^ influx through NMDARs on SG neurons of the Vc.

### 3.5. Effects of FCX on the Ca^2+^ Signaling

**Figure 8:**
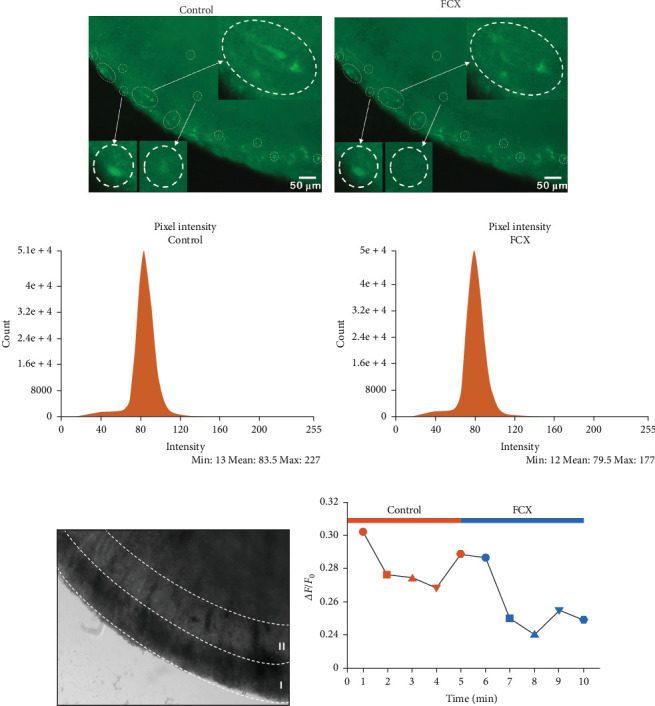
Effect of fucoxanthin (FCX) on Ca^2+^ signals on the trigeminal subnucleus caudalis (Vc) region. (a, b) Representative fluorescence images of brain slices stained with Fluo-3 AM depicting Ca^2+^ transients in the control and FCX treatment group, respectively. (c, d) Histograms of the intensity distribution showing the mean pixel intensity of the laminae I–II region measured after 3 min in the absence and presence of FCX (60 μM), respectively. (e) Microscopy image of brain slice including the laminae I–II region. (f) A time intensity histogram indicating the decrease of fluorescence intensity by the application of FCX compared to the control.

**Figure 9:**
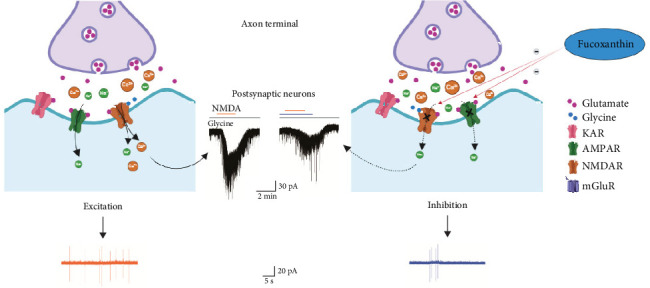
Schematic diagram illustrating the molecular mechanism underlying the inhibitory effect of fucoxanthin on glutamate excitotoxicity. Following release from presynaptic nerve terminal, glutamate binds to ionotropic glutamate receptors (iGluRs) (*N*-methyl-D-aspartic acid [NMDA] receptors [NMDARs], α-amino-3-hydroxy-5-methyl-4-isoxazolepropionic acid [AMPA] receptors [AMPARs], and kainic acid receptors [KARs]) and metabotropic glutamate receptors (mGluRs) on the membrane of postsynaptic neurons. Upon binding, the receptors initiate multiple responses, including membrane depolarization, activation of intracellular messenger cascades, and regulation of protein synthesis (not shown). After partial depolarization of the postsynaptic membrane, both glutamate and glycine attach simultaneously to NMDARs. The release of voltage dependent Mg^2+^ causes the NMDA channel to open and Ca^2+^ to flow into the postsynaptic neuronal cell. Fucoxanthin selectively regulates Ca^2+^ response through the NMDARs and thereby inhibits NMDAR-induced response on SG neurons of the Vc.

In the final step of experiments, to confirm the inhibitory effect of FCX on Ca^2+^ signal in the Vc region, we conducted fluorescence Ca^2+^ imaging within transverse brainstem slices.  Figure 8a,b depicts the fluorescence image in the control and FCX treatment group at three different regions of interest of acute brain slices. The fluorescence intensity of the laminae I–II region in the presence of FCX appeared to be weakened at each point. Specifically, the mean fluorescence intensity per pixel was reduced from 83.5 to 79.5 a.u. at 3-min frame in the absence and presence of FCX (60 μM), respectively, as shown in Figure 8c,d. Hence, the time intensity histogram (Figure 8f) revealed a decrease in the relative fluorescence intensity of the FCX group compared to the control at 1-min intervals, indicating that FCX could partially inhibit the Ca^2+^ response.  Figure 9 illustrates the inhibitory effect of FCX on glutamate excitotoxicity through regulating Ca^2+^ response in SG neurons of the Vc.

## 4. Discussion

Our study demonstrates for the first time the ability of FCX to inhibit iGluR hyperactivity electrophysiologically in the SG of the Vc. The suppressive effect of FCX on iGluRs was insensitive to the coapplication of TTX, Picro, and Stry, showing that FCX might function directly on iGluRs of postsynaptic SG neurons. Additionally, the FCX blockade of NMDA-mediated response involved the modulation of Ca^2+^ response without affecting Na^+^ ion. The Ca^2+^-dependent fluorescence intensity of brain slice was reduced in the presence of FCX. Consistent with potent efficacy of FCX on regulating excitatory transmissions, we also showed that FCX significantly attenuated the spontaneous firing activity of SG neurons. Together, our study provides new evidence that inhibition of Ca^2+^ response is likely to be the molecular mechanism underlying the neuroprotective effect of FCX against the electrical excitability of SG neurons.

Glutamate, the primary amino acid neurotransmitter of the CNS, plays a key role in both physiological (learning and memory) and pathological processes, namely, excitotoxicity, through iGluRs. iGluRs act as ionic channels following GluR binding, namely, NMDAR, AMPAR, and KAR [36]. It is well-known that prolonged exposure of neurons to glutamate results in the overactivation of NMDARs or AMPARs and a postsynaptic influx of Ca^2+^ up to cytotoxic level [5]. Interestingly, consistent with earlier observations, we detected that FCX elicited suppressive effect on glutamate-induced responses of SG neurons tested [21]. Emerging literature has indicated neuroprotective capacity of FCX in multiple experimental models of neurodegenerative disorders [18, 19]. More recently, one research demonstrated the cellular protective action of FCX against glutamate excitotoxicity via modulating parkin-mediated mitophagy pathway (also named ubiquitin-dependent mitophagy). FCX was proven to attenuate mitochondria dysfunction and cell death by promoting the parkin expression and increasing mitochondrial recruitment of autophagy regulators, receptors, and the autophagosome-related protein [21, 37]. Additionally, excitatory neurotransmission is mediated by both pre- and postsynaptically expressed iGluRs, which control neuronal network activity by modulating neurotransmitter release. Here, in the presence of TTX, Picro, and Stry, FCX still exhibited the inhibitory effect on glutamate-induced response, suggesting direct postsynaptic action of FCX on the iGluRs of SG neurons. Although this is the first time that we elucidate the neuroprotective effect of FCX on glutamate-triggered neuronal injury electrophysiologically, numerous compounds obtained from marine algae have exhibited powerful protective activity in various in vitro models. For example, fucoidan, a sulfated polysaccharide extracted from brown macroalgae, was noted to inhibit NMDA-mediated neurotoxicity in hippocampus [38] as well cortical neurons [39]. Likewise, several orange–red carotenoids in brown algae, for example, astaxanthin, exerted neuroprotection against excitotoxicity by modulating iGluRs, inhibiting intracellular Ca^2+^ elevation, and reactive oxygen species, as reported in the PC12, SH-SY5Y cells [22], hippocampal neurons [36], and primary cortical neuronal culture [23]. Hence, our study may provide a novel basis for further research on the application of FCX or other natural cytoprotective drugs.

It is worth mentioning that the molecular mechanism of FCX in the modulation of neuronal excitotoxicity has been previously revealed in a broad range of pathways. For example, FCX augmented neuronal survival against secondary injury and increased antioxidant enzymes via promoting Nrf2/ARE and Nrf2-autophagy signaling in several models of brain injury [17, 40]. Correspondingly, earlier studies have proven the FCX-mediated protection from cellular apoptosis induced by H_2_O_2_ or amyloid-β oligomer through the activation of prosurvival PI3K/Akt pathway and repression of proapoptotic ERK pathway [37, 41]. In the present study, the application of FCX significantly inhibited NMDA-induced responses in a concentration-dependent manner. In addition, such suppressive effect of NMDA currents by FCX was repeatable, implying that FCX had no effect on the desensitization of NMDARs. Given that there is substantial cross-talk between Ca^2+^ signaling and glutamate neurotoxicity [35, 42], we tried to identify the mechanism of FCX activity on the NMDAR inhibition on SG neurons. It is evident that NMDARs have high permeability to Ca^2+^. An aberrant change of cytoplasmic Ca^2+^ concentration via NMDARs may activate irreversible secondary sustained Ca^2+^ signaling cascades that lead to excitotoxic cell death [34]. Eventually, we examined the FCX action on NMDAR-mediated responses in the Ca^2+^- and Na^+^-free solutions to detect the relationship between FCX and Ca^2+^/Na^+^ influx via NMDARs. Our data indicate that NMDAR stimulation induced the bulk of Ca^2+^ transients from extracellular space. In the absence of Ca^2+^, the suppressive effect of FCX on NMDARs was totally blocked. By contrast, NMDAR currents continued to be inhibited by FCX in the Na^+^-free solution. This suggests the involvement of Ca^2+^ ion from the external space in the FCX inhibition of the NMDAR activation on SG neurons of the Vc. This result is in agreement with a preceding study, where FCX treatment was reported to prevent the increase of intracellular Ca^2+^ induced by Aβ, as measured by Fluo-3 AM [43]. Fucoidan administration attenuated intracellular Ca^2+^ concentration through the selective blockade of NMDARs in cortical neurons and L-type Ca^2+^ channels in hippocampal neurons [39]. Similarly, astaxanthin also revealed the inhibitory effect on mitochondrial reactive oxygen species formation and excessive Ca^2+^ rise induced by NMDARs [22, 36]. Other immunohistochemical reports demonstrated that astaxanthin suppressed iGluR-induced massive Ca^2+^ signaling by decreasing the mRNA expression levels of NMDA (GluN1) [22, 35]. It has been widely accepted that the use of Ca^2+^ indicators, including Fluo-3 AM, provides a reliable method to characterize Ca^2+^ transients in the activity of neurons in the CNS [44]. Notably, the regulatory effect of FCX on Ca^2+^ response was confirmed by the decrease in the fluorescence intensity in the presence of FCX compared to the control in this study [43]. Altogether, our findings demonstrate that the potential neuroprotective effects of FCX may partially involve the regulation of Ca^2+^ homeostasis. However, further research is required to identify clearly how FCX selectively regulates Ca^2+^ response through NMDARs.

It is well-known that glutamate-mediated cytotoxicity is a Ca^2+^-associated process. A rapid Ca^2+^ influx in response to glutamate treatment can result in an increase of intracellular Ca^2+^ concentration up to cytotoxic levels. Prolonged disturbances in the Ca^2+^ homeostasis may trigger major downstream signaling pathways of apoptosis, including the promotion of ER stress, mitochondrial dysfunction, and activation of calpain [22, 45]. Firstly, increased Ca^2+^ ions may severely impair ER function, activate apoptotic factors which in turn cause neuronal damage. ER stress has been involved in an increase in proapoptotic Bcl-2 family proteins (Bax and Bak) and a decrease in antiapoptotic Bcl-2 family proteins (Bcl-2 and Bcl-xL). Alteration in these protein levels results in cell death and has been indicated in the progression of neurodegenerative disorders [45]. A previous study has showed that FCX protected cells against H_2_O_2_-induced apoptosis by elevating Bcl-2 expression and inhibiting Bax expression. This protective action was accompanied by the downregulation of apoptosis promoting mediators (B-cell lymphoma-2-associated protein, caspase-9, and caspase-3) [46]. Similarly, FCX was reported to reduce the expression levels of genes encoding apoptotic proteins (caspase-3 and Bax), exhibiting the neuroprotective effect in H_2_O_2_- and Aβ_25-35_-induced C6 cell damage [47]. Besides, FCX pretreatment significantly inhibited ER stress and nucleus pulposus cell death in vitro and delayed the development of intervertebral disc degeneration in vivo [48]. Secondly, FCX was shown to protect mitochondria against AD- and PD-related pathology in vitro and in vivo. Ca^2+^ activates opening of mitochondria permeability transition pore (mPTP), a large nonselective channel. This activity can trigger apoptosis via mitochondrial membrane depolarization, adenosine triphosphate (ATP) depletion, and mitochondrial swelling. FCX inhibits Aβ-mediated upregulation of Bax, thereby helping maintain the mitochondrial membrane integrity [43]. Besides, FCX treatment protected mitochondria against reactive oxidative stress in vitro and increased DJ-1 expression, an oxidative stress sensing protein, in the hippocampus in vivo [49]. Thirdly, calpain, a Ca^2+^-dependent cysteine protease that cleaves various biologically important proteins and regulates glutamate-induced apoptotic events, can cause degradation of Ca^2+^ regulatory proteins (SERCA and Ca^2+^ channel proteins) and activate the release of Ca^2+^ from ER [50]. Oral administration of FCX also reduced the activity of calpain-1 on ultraviolet A-induced photoaging in vivo [51]. Thus, the inhibition of Ca^2+^ influx and calpain activity by FCX contributes to its protective effects against glutamate-induced neurotoxicity.

Even though NMDARs, AMPARs, and KARs belong to subfamilies of iGluRs due to their pharmacological and structural similarities, they yielded specific vulnerabilities to FCX. Of note, we found that FCX inhibited AMPAR activation just in over 70% SG neurons tested and unaffected KA-induced electrical response. A known difference between the NMDARs and AMPARs/KARs is the Ca^2+^ permeability of receptors. These findings are confirmed in previous reports that NMDARs are uniformly Ca^2+^-permeable, whereas the Ca^2+^ permeability of AMPARs depends on the presence of their subtypes. AMPAR is an oligomeric assembly of four subtypes designated GluA1–GluA4. Earlier studies have suggested that when AMPARs are assembled from the combination of GluR subunit 1, 3, and 4 (GluA1, 3, and 4), the receptors are highly permeable to Ca^2+^ [5]. Indeed, Ca^2+^-permeable AMPARs play an essential role in synaptic plasticity, learning, and disease [52]. Furthermore, the distinctive inhibitory effects of FCX on iGluR stimulation were similar to previous observations, where fucoidan or astaxanthin treatment alleviated excitatory transmissions in the order NMDA > AMPA > KA. The astaxanthin-induced suppression of Ca^2+^ influx through NMDARs and AMPARs involved the loss of the protein expression of NMDA (GluN1) and AMPA (GluA2). In contrast, these marine compounds unaffected the Ca^2+^ response under KA activation and unchanged the protein expression level of KARs [23, 38]. This finding corroborates with Ca^2+^-impermeable property of KARs. Overall, FCX suppresses NMDAR and AMPAR activity, but not KARs on SG neurons, suggesting the relationship of FCX and Ca^2+^ ion regulation via NMDARs and AMPARs. While most AMPARs in the brain contain the edited GluA2 subunit and are Ca^2+^-impermeable, AMPARs lacking GluA2 or containing unedited GluA2 subunit are still expressed in both neurons and glia, including dorsal horn neurons in culture and in situ, GABAergic neurons of lamina II, and hippocampal neurons [40]. Although Ca^2+^ influx via AMPARs is less significant than that via NMDARs, the location and kinetics of this influx enable it to perform different cellular functions. Ca^2+^-permeable AMPARs may be identified by the combination of various techniques, such as Ca^2+^ imaging, electrophysiology, and RNA editing assay [53]. As a result, future research is essential to observe the presence of Ca^2+^-permeable AMPARs in the Vc region.

As the activation of NMDARs is crucial for central sensitization concerning chronic pain, NMDAR antagonism is considered as a powerful analgesic [54]. However, most efforts into the therapeutic benefits of NMDAR antagonists have exhibited unsatisfactory results due to their limited clinical tolerability. As a result, novel medications of this target should carefully reduce NMDAR functions without eliminating them. Although there is little clear evidence demonstrating the antinociceptive effect of FCX on the SG of the Vc, a pivotal site in modulating nociceptive transmission, we believe that iGluR antagonism of FCX via regulating Ca^2+^ response is capable of protecting SG neurons from excitotoxicity.

## 5. Conclusions

The present study demonstrates that FCX inhibits NMDAR- and AMPAR-induced glutamatergic transmission by regulating Ca^2+^ response in SG neurons of the Vc. Moreover, FCX significantly suppresses the spontaneous firing activity on SG neurons, suggesting the SG neuroprotective effect of FCX. However, further research is necessary to investigate how FCX controls the specific receptors.

## Data Availability

The data that support the findings of this study are available from the corresponding author upon reasonable request.

## Nomenclature

AMPA: α-Amino-3-hydroxy-5-methyl-4-isoxazolepropionic acid
CNS: Central nervous system
FCX: Fucoxanthin
GABA: γ-Aminobutyric acid
iGluRs: Ionotropic glutamate receptors
KA: Kainic acid
mGluRs: Metabotropic glutamate receptors
NMDA: *N*-Methyl-D-aspartic acid
ROI: Region of interest
SG: Substantia gelatinosa
TTX: Tetrodotoxin
Vc: Trigeminal subnucleus caudalis.
